# The coping strategies during medical education predict style of success in medical career: a 10-year longitudinal study

**DOI:** 10.1186/s12909-016-0706-1

**Published:** 2016-07-22

**Authors:** Małgorzata Tartas, Maciej Walkiewicz, Waldemar Budziński, Mikołaj Majkowicz, Krzysztof Wójcikiewicz, Agata Zdun-Ryżewska

**Affiliations:** Faculty of Psychology, Medical University of Gdańsk, Tuwima 15 Street, 80-210 Gdańsk, Poland; Polish Chamber of Physicians in Gdansk, Śniadeckich 33 Street, 80-204 Gdańsk, Poland

**Keywords:** Coping, Longitudinal study, Job satisfaction, Medical students, Medical doctors, Professional success, Burnout, Quality of life

## Abstract

**Background:**

The stress associated with the physician’s work is generally acknowledged and is related to well-being and life satisfaction. The presented study was designed to extract the role of coping strategies in identifying differentiated styles of success in a medical career during medical education.

**Methods:**

The participants were examined when they applied to medical school and each subsequent academic year. The final study took place four years after graduation. The baseline questionnaire measured coping strategies. The follow-up questionnaire consisted of measures of: quality of life, work stress and burnout, satisfaction with medicine as a career, and professional competency.

**Results:**

Based on coping strategies assessed during admission and preclinical years of medical study, some aspects of success in the participants’ future medical career can be predicted. Students who take action and deal directly with a problem, neither accept resignation, nor reduce tension by expressing feelings would most probably resist future burnout. However, despite the fact that they obtain the highest quality of life or earn the highest income they would be, at the same time, the least satisfied with chosen career, as well as being more likely to be characterised by a low level of competence.

**Conclusions:**

Assessment of coping strategies at the beginning of medical education could be taken into consideration as an instrument to diagnose a specific trend in physicians’ career development.

## Background

The stress associated with medical doctor’s work is generally acknowledged. Physicians have an increased prevalence of certain mental health problems as compared to the general population [1–4]. During their education they suffer from excessive levels of stress, and distressful occurrences are mostly related to the medical training, rather than to individual psychological qualities [5–8]. The first postgraduate years are particularly stressful. Young doctors may have unrealistic expectations of their future work, and supposable are not enough prepared to cope with the associated stress [9–12]. There are limited research data available on the nature of the possible correlation between an undergraduate medical education and the incidence of later professional and psychological problems. It may be that no optimal development of constructive coping strategies, critical thinking, self-reflection, or empathetic reflection takes place at school [13, 14]. During study the nature of the stressors changes and the medical students may experience a great number of emotional problems and probably need more support [15, 16]. This causes some coping strategies to be more effective in the different phases of professional development. Students who have begun school and who use avoidant coping strategies, are found to be on risk of evolving mental health problems later [17]. In dealing with stressors connected with medical school, students of the preclinical years use more problem-solving and self-blame strategies, and confront coping strategies less [18].

The literature has provided data suggesting that there is no unarguable model that would describe job success in a medical field. The longitudinal study ‘Social Diagnosis’, which presents information about quality of life of Polish doctors, was an inspiration in developing our model of success in the medical career [19]. The ‘Social Diagnosis’ research is one of the largest longitudinal psychological studies of quality of life in the population of a specific country. Authors return to the same families every few years (2000, 2003, 2005, 2007, 2009, 2011, 2013, 2015). In 2015, *n* = 24,324 (the population of Poland is approximately 38 million). The results of this study also characterise life satisfaction and well-being in Polish physicians, comparing them to other professions [19].

In our research, we devised a specific model based on life satisfaction and well-being, with reference to another model of career success, derived from the literature, to include such complementary factors as job success (performance, happiness at work) and material success [20]. In our model we included work stress and the tendency to burnout as expression of difficulty or defeat in response to career expectations, and as significant measures of success in a medical career [21–24].

In this longitudinal research project (1999–2009), we measured predictors of success in a medical career. The results highlight success as the consequence of personality characteristics rather than as a simple effect of the education process [25]. Coping strategies as an independent variable were followed by academic achievement; sense of coherence, anxiety, depression, value system and need for social approval. The dependent variables group consisted of: satisfaction with medicine as a career, work stress and vulnerability to burnout, quality of life and postgraduate medical competence. The data stated that academic achievement just explained professional competence, while work satisfaction and tendency to burnout were related to job performance, the quality of life was linked to these by psychological profile expressed by: depression, anxiety, sense of coherence, as well as coping strategies. This supported our thinking that success in a medical career is rather the effect of the level of personality structure integration, and not just the simple result of the educational development [25].

In the next stage, in order to improve the practical applicability of the presented data, we identified styles of success in the medical career using the cluster analysis procedure. We managed to indicate three styles, the first of which were those physicians who were ‘Committed - satisfied with career’. They had the lowest postgraduate medical competence but in the same time they manifest the highest level of work satisfaction. However, they experienced the highest tendency to burnout (and work stress) and were not content with life.

The second style applies to physicians who displayed low competence. They manifest the lowest level of work satisfaction as well as the lowest level of burnout. Their quality of life at this point was the highest. Additionally, they were the most satisfied with life, and they also get the highest financial gratification. Surprisingly, these medical doctors were the least committed to the profession while seeming to derive the most benefit from it. This group we called ‘Clever - satisfied with life’.

**Fig. 1:**
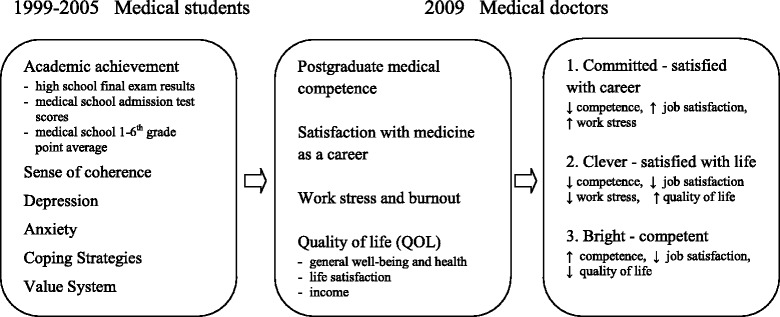
Model of success predictors and markers of success four years after graduation

The third cluster was made up of the most competent physicians, who were, however, dissatisfied with medicine as a career. Although they represented a moderate level of burnout and their quality of life was the lowest. They also achieved the lowest income and they were the most dissatisfied with life, generally. We called them ‘Bright - competent’ [26, 27] (see Fig. 1, Table 1).

**Table 1:** Descriptions of the three styles of success in a medical career dimensions: cluster analysis (Bineary Euclidean distance measure and Ward’s linkage method)

	1. Committed satisfied with career *n*=14	2. Clever satisfied with life *n*=20	3. Bright unsatisfied *n*=16			Committed vs Clever	Committed vs Bright	Clever vs Bright
	M ± SD	M ± SD	M ± SD	F (2;47)	P	Tukey’s	post-	hoc test
Postgraduate medical competence	−0.62 ± 0.61	−0.50 ± 0.53	1.17 ± 0.61	48,256***	<0.001	0,53	<0.001***	0,00**
Satisfaction with medicine as a career	0.95 ± 0.68	−0.47 ± 0.47	−0.24 ± 0.85	20,180***	<0.001	<0.001***	<0.001***	0,31
Work stress and burnout	0.33 ± 0.48	−0.27 ± 0.43	0.07 ± 0.45	7,365***	<0.001	<0.001***	0,13	0,03*
Quality of Life General well-being	−0.29 ± 0.81	0.49 ± 0.55	−0.35 ± 0.60	9,499***	<0.001	<0.001***	0,80	<0.001***
Quality of Life Life satisfaction	−0.60 ± 0.78	0.76 ± 0.45	−0.89± 0.86	29,090***	<0.001	<0.001***	0,26	<0.001***
Quality of Life Income	3.57 ± 0.51	3.80 ± 0.41	2.63 ± 0.89	16,803***	<0.001	0,31	<0.001***	<0.001***

Source: Tartas M, Walkiewicz M, Budziński W, Majkowicz M, Wójcikiewicz K. The sense of coherence and styles of success in the medical career: a longitudinal study. BMC Med Educ. 2014;14:254. doi:10.1186/s12909-014-0254-5

*p<0.05; **p<0.01; ***p<0.001

The presented study was designed to extract the role of coping strategies in identifying differentiated styles of success in a medical career during medical education.

## Methods

### Participants

The first stage of the study took place a few days before the admission (June 1999). All candidates who had applied to the Medical University of Gdańsk, Poland received a questionnaires (*n* = 365 of 940, 39 % response rate). Only those who passed the admission test were taken into consideration for the purposes of our project (*n* = 320). The procedure was constantly repeated at the end of each academic year (2000–2005). Four years after graduation we cooperated with institutions responsible for postgraduate medical education. The Medical Examination Centre in Poland provided access to the examination results of postgraduates for 268 identified medical doctors. Additionally we cooperated with the Polish Chamber of Physicians in Gdańsk, where we received the addresses of 255 physicians who had participated in the first part of the project. The respondents received a letter sent by the Chamber of Physicians with a request to fill in an electronic questionnaire. The response rate was *n* = 54. The mean age of respondents was 29.5 ± 0.8 years (July 2009), 69 % female. The response rates are presented in Table 2:

**Table 2:** The response rates

1999 - Admission	n=178
2000 - First year of medical study	n=178
2001 - Second year of medical study	n=129
2002 - Third year of medical study	n=127
2003 - Fourth year of medical study	n=121
2004 - Fifth year of medical study	n=58
2005 - Sixth year of medical study	n=138
2009 - Four year after medical study	n=54

### Measures

#### Independent variables

The coping strategies at admission and during medical study were measured by Coping Responses Inventory (Moos). The first four subscales assess approach coping responses: ‘Logical Analysis’ (cognitive attempts to understand a stressor and its consequences), ‘Positive Reappraisal’ (cognitive attempts to construe and restructure a problem in a positive way), ‘Guidance and Support Seeking’ (behavioural attempts to seek information, guidance, or support) and ‘Problem Solving’ (behavioural attempts to take action and deal directly with a problem). The second four subscales assess avoidance coping responses: ‘Cognitive Avoidance’ (cognitive attempts to avoid thinking about a problem), ‘Acceptance or Resignation’ (cognitive efforts to deal with a problem by accepting it), ‘Seeking Alternative Rewards’ (behavioural attempts to cope by finding substitute activities or sources of satisfaction), and ‘Emotional Discharge’ (behavioural efforts to reduce tension by expressing negative feelings) [28–30].

#### Dependent variables

We identified three styles of success in a medical career based on significant differences between them in terms of postgraduate medical competence, satisfaction with medicine as a career, work stress and burnout and quality of life [26, 27] (see Fig. 1, Table 1).

The postgraduate medical competence was measured by the examination results in the State Examination for Medical Doctors, supplied by the Medical Examination Centre. In Poland this exam is administered during the postgraduate internship and it is required to gain a license to practice medicine. The results establish whether or not further specialization in medicine will be possible. The exam is organised by the Medical Examination Centre in Łódź each spring and autumn, starts simultaneously in eleven districts and is a multiple-choice test. The second aspect of success - burnout - was measured by the Maslach Burnout Inventory (MBI), which has three sub-scales: Emotional Exhaustion, Depersonalisation, and Personal Accomplishment [31].

The third parameter of success in medical career - satisfaction with medicine as a career - was measured by a self-designed survey based on the Cantril’s Scale method, where 1 means “very low” and 10 means “very high” (Cronbach’s alpha = 0.80; *r* = 0.67).

The quality of life (QoL) - was measured by a questionnaire derived from ‘Social Diagnosis’ [19].

Quality of life in our research model consisted of:

A) General well-being and health consists of two questions:

“Thinking about your life over the last two weeks, would you say that it has been: unhappy; not happy; quite happy; very happy”.

“Thinking about the whole of your life, would you say that it has been: awful; unhappy; not very successful; neither good nor bad; pretty good; successful; great”.

(Cronbach’s alpha = 0.74, Discriminatory power *r* = 0.40).

B) Life satisfaction. We asked 22 questions about different aspects of human life: social, financial, surroundings and health:

“Please assess these individual aspects of your life, and say how satisfied you are by them: 1 - very satisfied; 2 - satisfied; 3 - quite happy; 4 - quite dissatisfied; 5 - dissatisfied; 6 - very dissatisfied; 0 - not applicable”.

“Children; the ability to satisfy one‘s nutritional needs; marriage; your educational level; your state of health; future prospects; relationships with close family members; sexual life; relations with colleagues and superiors; safety in the place of residence; relationships with friends (or group of friends); place of residence; your life achievements; level of available goods and services; housing conditions; manner of spending leisure time; financial situation of the family; current family income; work; moral standards in your environment; the situation in the country” (Cronbach’s alpha = 0.83; Discriminatory power *r* = 0.25).

C) Size of income.

### Statistics

All the statistical methods we used were exploratory in nature, so that the use of subsequent analyses is dependent on the previous outcomes. In the earlier phase of our research, cluster analysis was used to identify styles (clusters) of success. In the present study, ANOVA analysis of variance was used to determine the differences between clusters during studies, cross checked with the coping strategies. In order to reduce this error in the third step, we used discriminant analysis (with the backward method using Ward’s estimate). The predictors were variables from ANOVA, and the dependent variables were styles of success (clusters).

## Results

We explored the relation between specificity of coping strategies during medical education and the styles of success in a medical career. In our previous research, we have established three different styles of success [26, 27] (see Fig. 1, Table 1).

We found significant differences in: ‘Problem Solving’, Approach Coping strategy, ‘Acceptance or Resignation’ and ‘Emotional Discharge’ and Avoidant Coping strategy only at the beginning of the education process.

The ‘Problem Solving’ strategy during admission and the first year of medical studies was the highest in the ‘Clever’ styles (admission M = 54.30, first year M = 56.60) and the lowest in the ‘Committed’ style (admission M = 47.29, first year M = 49.00). The level of this strategy was significantly higher in ‘Clever’ than ‘Committed’.

In the same moment of education (admission and first year of study), another strategy, ‘Acceptance or Resignation’ was the lowest in ‘Clever’ style (admission M = 44.00, first year M = 44.80), but the highest in ‘Bright’ group (admission M = 51.38, first year M = 50.63). However, the ‘Clever’ have a significantly lower tendency to accept or resign than the ‘Bright’ only during admission.

The situation changed in the second year, when ‘Emotional Discharge’ makes a significant difference, as the level of this strategy is the lowest in ‘Clever’ (admission M = 47.29, first year M = 49.00).

The ‘Emotional Discharge’ strategy during first year of medical study was the highest in the ‘Bright style (first year M = 65.25) and the lowest in the ‘Clever’ style (first year M = 57.00). This situation changed in the second year when ‘Emotional Discharge’ makes a significant difference, as the level of this strategy is the highest in ‘Committed’ style (second year M = 62.57), but still the lowest in ‘Clever’ style (second year M = 56.70) (see Table 3).

**Table 3:** Means (± standard deviation) for groups representing the three styles of success in the medical career (year 2009), in terms of coping strategies at admission and during medical school (years 1999–2005)

	Dependent variables (2 0 0 9)								
Independent variable (1 9 9 9 – 2 0 0 5)	Committed ↓ competence ↑ job satisf. ↑ work stress	2. Clever ↓ competence ↓ job satisf. ↓ work stress ↑ QoL	3. Bright ↑ competence ↓ job satisf. ↓ QoL				Committed vs Clever	Committed vs Bright	Clever vs Bright
	M±SD	M±SD	M±SD	F (2;47)	P	Eta^2^	Tukey’s	post-	hoc test
Approach Coping									
Logical Analysis (cognitive)									
1999 - Admission	53.43±2.65	53.60±5.53	52.88±5.83	0.097	0.907				
2000 - First year of medical study	54.14±3.57	52.20±7.41	52.38±5.86	0.481	0.621				
2001 - Second year of medical study	54.71±5.20	52.80±5.67	54.25±4.37	0.657	0.523				
2002 - Third year of medical study	54.71±5.20	52.30±6.62	54.25±4.37	0.926	0.403				
2003 - Fourth year of medical study	52.86±2.85	52.60±6.67	53.63±4.29	0.187	0.830				
2004 - Fifth year of medical study	54.71±6.02	52.30±6.76	54.38±3.69	0.925	0.404				
2005 - Sixth year of medical study	55.71±6.22	52.30±6.76	54.38±3.69	1.505	0.232				
Positive Reappraisal (cognitive)									
1999 - Admission	51.14±6.46	51.20±5.58	48.00±8.50	1.164	0.321				
2000 - First year of medical study	49.29±6.97	52.20±6.88	47.75±8.47	1.668	0.200				
2001 - Second year of medical study	52.00±6.97	51.30±7.06	50.88±6.96	0.098	0.907				
2002 - Third year of medical study	52.00±6.97	51.30±6.79	50.88± 6.96	0.101	0.904				
2003 - Fourth year of medical study	50.57±6.25	52.20±6.91	51.25±6.88	0.251	0.779				
2004 - Fifth year of medical study	53.00±5.71	50.40±6.60	51.25±6.88	0.673	0.515				
2005 - Sixth year of medical study	52.43±5.63	50.40±6.60	51.25±6.88	0.408	0.667				
Guidance and Support Seeking (behavioral)									
1999 - Admission	54.71±10.01	54.30±8.75	54.63±8.50	0.010	0.990				
2000 - First year of medical study	54.71±10.75	55.60±6.90	56.50±7.83	0.169	0.845				
2001 - Second year of medical study	53.14±9.74	55.60±8.84	56.25±6.87	0.547	0.582				
2002 - Third year of medical study	53.14±9.74	57.90±6.45	56.25±6.87	1.613	0.210				
2003 - Fourth year of medical study	52.86±9.82	58.40±5.70	55.00±5.24	2.745	0.075	0,12	0,07	0,68	0,32
2004 - Fifth year of medical study	56.00±10.26	56.50±6.51	55.38±5.26	0.102	0.903				
2005 - Sixth year of medical study	57.14±11.00	56.50±6.51	56.00±5.96	0.079	0.924				
Problem Solving (behavioral)									
1999 - Admission	47.29±6.92	54.30±8.74	53.25±4.43	4.382	0.018*	0,19	0,02*	0,07	0,90
2000 - First year of medical study	49.00±8.06	56.60±7.88	55.13±5.25	4.871	0.012*	0,21	0,01*	0,06	0,82
2001 - Second year of medical study	51.86±7.22	54.60±7.14	52.50±5.24	0.824	0.445				
2002 - Third year of medical study	51.86±7.22	53.80±7.92	52.50±5.24	0.348	0.708				
2003 - Fourth year of medical study	50.71±6.85	54.80±6.96	52.13±5.15	1.806	0.175				
2004 - Fifth year of medical study	54.00±6.61	53.80±7.24	53.00±5.22	0.105	0.901				
2005 - Sixth year of medical study	54.43±6.56	53.80±7.24	53.38±5.61	0.097	0.908				
Avoidant Coping									
Cognitive Avoidance (cognitive)									
1999 - Admission	51.71±10.69	50.20±6.32	48.38±6.34	0.694	0.505				
2000 - First year of medical study	51.29±10.88	50.50±5.11	50.75±8.88	0.038	0.963				
2001 - Second year of medical study	47.29±9.81	50.80±5.23	50.63±8.23	1.006	0.373				
2002 - Third year of medical study	47.29±9.81	52.20±5.63	50.63±8.23	1.647	0.204				
2003 - Fourth year of medical study	48.57±8.48	50.90±5.96	52.38±6.87	1.107	0.339				
2004 - Fifth year of medical study	46.86±7.49	49.80±6.44	51.50±7.38	1.648	0.203				
2005 - Sixth year of medical study	45.86±8.03	49.80±6.44	50.88±8.04	1.877	0.164				
Acceptance or Resignation (cognitive)									
1999 - Admission	48.86±4.59	44.00±5.29	51.38±7.90	6.863	0.002**	0.29	0.07	0.50	0.00*
2000 - First year of medical study	49.57±5.37	44.80±5.44	50.63±8.67	3.976	0.025*	0.17	0.11	0.90	0.03
2001 - Second year of medical study	43.57±5.43	44.00±4.18	47.63±5.56	3.164	0.051	0.13	0.97	0.08	0.09
2002 - Third year of medical study	43.57±5.43	44.50±4.80	47.63±5.56	2.579	0.087	0.11	0.87	0.10	0.19
2003 - Fourth year of medical study	45.29±7.25	44.50±5.11	48.63±4.62	2.544	0.089	0.11	0.92	0.25	0.09
2004 - Fifth year of medical study	45.14±3.11	44.50±6.84	48.00±5.14	1.954	0.153				
2005 - Sixth year of medical study	43.86±5.27	44.50±6.84	48.63±4.62	3.188	0.050	0.14	0.95	0.07	0.10
Seeking Alternative Rewards (behavioral)									
1999 - Admission	54.57±5.29	56.50±6.76	54.38±7.15	0.586	0.560				
2000 - First year of medical study	55.86±5.07	58.40±6.73	56.00±6.85	0.906	0.411				
2001 - Second year of medical study	53.29±5.37	55.90±5.55	52.50±6.87	1.620	0.209				
2002 - Third year of medical study	53.29±5.37	56.30±4.57	52.50±6.87	2.321	0.109				
2003 - Fourth year of medical study	52.86±5.36	56.30±3.37	54.25±7.24	1.740	0.187				
2004 - Fifth year of medical study	54.86±5.13	56.10±6.23	52.38±7.10	1.601	0.212				
2005 - Sixth year of medical study	55.14±5.96	56.10±6.23	53.00±7.36	1.019	0.369				
Emotional Discharge (behavioral)									
1999 - Admission	61.43±11.66	57.00±6.16	65.25±6.88	4.488	0.016*	0.19	0.28	0.42	0.01*
2000 - First year of medical study	61.86±11.29	58.20±4.40	62.25±6.34	1.615	0.210				
2001 - Second year of medical study	62.57±9.91	56.70±5.75	61.50±5.59	3.425	0.041*	0.15	0.06	0.91	0.12
2002 - Third year of medical study	62.57±9.91	57.90±6.75	61.50±5.59	1.894	0.162				
2003 - Fourth year of medical study	62.29±11.47	57.30±6.52	60.75±5.31	1.805	0.176				
2004 - Fifth year of medical study	62.29±12.10	59.40±4.52	61.88±5.35	0.748	0.479				
2005 - Sixth year of medical study	60.14±14.64	59.40±4.52	61.50±5.59	0.255	0.776				

*p<0.05; **p<0.01; ***p<0.001

## Discussion

According to the presented data, we would underline that admission and the beginning of medical studies (first and second year) constitutes the moment coping strategies are significant factors in specific predictions of the future style of success in a medical career.

Our findings widely describe the main specific ways of coping in medical students, who, as a physicians, would not be in the risk group of burnout. The group was called ‘Clever - satisfied with life’. During medical studies, they use behavioural attempts to take action and deal directly with problems, however, they do not use cognitive efforts to accept or resign. They do not use behavioural efforts to reduce tension by expressing negative feelings. Later, as physician, they are resistant of burnout, have the highest quality of life and income, a low level of postgraduate competence, and the least satisfaction with their chosen career. Presumably, they are not overly engaged, and so have other life priorities and values. This is why they are not in the risk group of burnout. Conversely, in terms of coping, students, who, as physicians, are the most competent, have difficulties with governing their lives. They were called the ‘Bright - competent’. These students cope with stress by using cognitive efforts, in this case dealing with problems by accepting them. They also introduce behavioural efforts to reduce tension by directly expressing negative feelings. As physicians, they are likely to give up, e.g. towards the limitations of the curative process, but could bring up difficulties in the relationships because they are more likely to express emotions than other groups. The last style called ‘Committed - satisfied with career’, the most involved in their work, have the highest level of work stress as well as vulnerability to burnout. During studies they have the lowest level of problem solving competency.

We are most easily able to predict belonging to the ‘Clever’ group, according to specific coping at admission and the first and second year of study, while differentiating between the other two groups, ‘Bright’ and ‘Committed’, is not as precise. ‘Bright’ could be differentiated in the same moment only based on two strategies and ‘Committed’, due to their limitation, which is a deficit of specific strategy at the admission and the first year.

Assessment of coping is not a perfect way to diagnose a specific tendency in medical career development. However, according to the presented data it could provide a background to job counselling, as well as a personal development process for medical students, starting from their admission. This could be identified as a strength of the presented study. Compared to other studies, our findings introduce long-term monitoring of the practical application of the coping concept in a medical career.

Although our study presents a range of serious limitations, e.g. the evidence does not propose an consistent clarification of the styles of success for the whole of the physician representation, it may add a valuable perspective to the understanding of the development of a medical career. Attempts to assess the link between styles of success and the coping process in medical training in the future, should be based on a larger research population, as it would be challenging to consider coping strategies in drop-outs from medical career.

## Conclusions

Coping strategies are of great importance to identify specific group of medical students and their way of career development. They are problem oriented, not very likely to resign neither nor to express negative feelings. What needs highlighting they are satisfied with their lives.

## Abbreviations

MBI, Maslach Burnout Inventory; QoL, quality of life
